# *Salmonella enterica* 13,23:i:- in a UK Broiler Hatchery: A Longitudinal Case Study of the Distribution of Resident Contamination and Its Control

**DOI:** 10.3390/microorganisms14081747

**Published:** 2026-08-08

**Authors:** Kate Newton, Andrew Wales, Christopher Nichols, Claire Oastler, Francesca Martelli, Robert Davies

**Affiliations:** 1 Department of Bacteriology, Animal and Plant Health Agency, Addlestone KT15 3NB, Surrey, UK; katharine.newton@apha.gov.uk (K.N.);; 2 Discipline of Comparative Biomedical Sciences, School of Veterinary Medicine, University of Surrey, Guildford GU2 7AL, Surrey, UK; a.wales@surrey.ac.uk

**Keywords:** *Salmonella*, hatchery, disinfection, biofilm, biosecurity

## Abstract

Hatcheries potentially can disseminate *Salmonella* through poultry production chains. This study describes the investigation and control of persistent *Salmonella enterica* serovar 13,23:i:- in a UK broiler hatchery. Over four years from 2015 to 2019, a broiler hatchery was sampled intensively seven times, yielding *Salmonella* from 361 of 2645 samples. The findings assisted biosecurity advice and the targeting both of cleaning and disinfection (C&D) and subsequent sampling rounds. Additional sampling was conducted to assess C&D and monitor antimicrobial resistance, and also among all the supplying breeder flocks. The large majority of the isolates were *Salmonella* 13,23:i:- and the degree of contamination increased along the process pathway. The egg areas and the setter incubators were the least contaminated, whilst the hatcher cabinets, the chick handling areas, and the waste and external areas yielded *Salmonella* most frequently. The likely resident serovar 13,23:i:- was not eradicated during this study, but diligent management and C&D enhancements and targeting, plus effective biosecurity, were eventually followed by a substantial reduction in *Salmonella* contamination. The percentage of positive samples per visit reduced to 2.3% from a mid-study peak of 23%. The antimicrobial resistance was varied, infrequent (detected in 4% of tested isolates), and did not show longitudinal trends. This study shows that long-term *Salmonella* hatchery contamination may be susceptible to targeted interventions, as informed by repeated sampling, provided that biosecurity is of a high standard and C&D (including biofilm disruption) has been optimised. Importantly, it also illustrates the long timescale over which interventions may be required to reduce resident *Salmonella* contamination.

## 1. Introduction

Salmonellosis is one of the most common gastrointestinal infections in humans, with over 77,000 confirmed cases in the European Union (EU) in 2023 [1]. Poultry is an important source for human salmonellosis [2,3,4], with broiler meat and table eggs being major vehicles [1]. Breeding flocks, feed mills and hatcheries act as potential focal points of *Salmonella* contamination in poultry production [5,6,7,8]. Hatcheries can disseminate *Salmonella* to commercial flocks [9,10,11,12,13,14], and this role can be amplified by the structure of the industry, with large integrated companies relying on relatively few supplying hatcheries. Hatchery *Salmonella* strains may contaminate consumer products [15], with risk-based modelling in Canada by Racicot et al. [16] suggesting that up to 4% of poultry meat salmonellosis cases are attributable to contamination from hatcheries.

Chicks can be infected with *Salmonella* by vertical transmission if the eggs are internally infected by certain serovars (such as *Salmonella* Enteritidis), by pseudo-vertical transmission if external egg contamination penetrates the shell, or via horizontal transmission during hatching [17,18,19]. To reduce external egg contamination, and thus the risk of *Salmonella* dissemination within hatcheries, in the United Kingdom (UK), eggs are usually disinfected by fogging before entering first-stage incubators (setters) [20]. 

Certain *Salmonella* strains can become resident in a hatchery, persisting as an environmental contaminant over indefinite cycles of egg incubation and hatching. Such contamination is usually focused in areas with surfaces that are difficult to clean and disinfect, such as hatchers and the associated ventilation ducting systems, egg and hatch waste processing areas and hatcher basket wash machines [21,22]. *Salmonella* can survive for years in materials such as hatcher fluff and dust, and it can persist in the hatchery without further incoming infected eggs [21,22,23]. Certain *Salmonella* serovars, such as *S.* Senftenberg, can be very persistent within incubators and the associated ducting, possibly because of their ability to form biofilms or a reduced susceptibility to desiccation or biocides [24,25,26,27]. Once resident, a *Salmonella* strain can be very difficult to eliminate and may become a long-term source of infection for newly hatched chicks [22,28]. 

Cox et al. [23] showed that hatching chicks are highly susceptible to *Salmonella* infection, making hatchery contamination especially risky. Chicks may ingest or inhale contaminated material within the hatchers [23,29] or from other chick processing areas inside the hatchery. Contamination can easily spread between chicks in hatcher baskets and within hatchers via dust, debris and fluff [29]. There may also be longitudinal carryover of *Salmonella* between hatchings if cleaning and disinfection (C&D) is ineffective. There may be further horizontal transmission during procedures in the chick processing areas. Thus, despite typically very low proportions of *Salmonella*-positive eggs entering hatcheries (0.01–0.05%), up to 9% of leaving chicks can be colonised by *Salmonella* [30]. Such chicks are then able to disseminate *Salmonella* to other batches during transit and when placed on farms [31].

*Salmonella* control depends upon disinfecting eggs effectively upon entry, although better protection against pseudo-vertical contamination is achieved by sanitising the eggs on the farm as soon after lay as possible [32,33]. Furthermore, there should be multiple barriers to *Salmonella* entry and spread, including good internal and external biosecurity, with carefully planned unidirectional workflows and air pressure gradients to counter the spread or recycling of contamination, plus thorough and effective C&D programs [20,33,34] validated by sensitive monitoring programs. Alternatively, the centralised process can be replaced by on-farm hatching to reduce the risk of disseminating contamination from different sources [35].

UK strains of *Salmonella enterica* subsp. *enterica* serovar 13,23:i:- (*S.* 13,23:i:-) are thought to be variants of *S.* Idikan [36], based on whole genome sequencing. *S.* Idikan is often associated with contaminated feed and mills, and it can also become persistent in hatcheries and on poultry farms [37]. *S.* 13,23:i:- was first isolated from broilers in the UK in 2009, and it has subsequently become widely established across the poultry industry, especially within the broiler sector [37]. In 2018, it was the most commonly isolated serovar from chickens and from compound poultry feed [38]. However, it does not appear to be widely established in the EU, accounting for less than 2% of *Salmonella* reports from broiler flocks between 2014 and 2016 [18], all of which were from the UK. Furthermore, it has not been among the top 20 EU broiler serovars in subsequent years [1]. There is also no evidence of its significant involvement in human cases [39,40].

The present report describes a four-year investigation triggered by the isolation from a UK broiler hatchery (via routine monitoring) of a *Salmonella* serovar of particular relevance to public health. This serovar was not recovered subsequently, and the investigation focused on C&D to address the repeated recovery of a perceived resident serovar, *S.* 13,23:i:-. Intensive sampling visits highlighted the areas that were particularly susceptible to contamination, enabling better targeting of C&D, as well as informing biocide selection and specific biosecurity advice.

In contrast to the existing *Salmonella* survey reports, overviews, and investigations, the current work focuses in detail on a hatchery with a particular, multifactorial contamination problem. It illustrates the strategy of detailed and repeated surveys and interventional work that may be needed to effectively suppress or eliminate persistent *Salmonella* contamination (whether of this serovar or of others) in a particular situation. It additionally demonstrates common ‘problem’ areas and shows that, in a single hatchery, several issues may need to be first identified and then addressed before control is achieved. Additional small studies on *Salmonella* recovery technique and longitudinal antimicrobial resistance are included.

## 2. Materials and Methods

### 2.1. Hatchery Operation

The hatchery was supplied by 60 broiler breeder farms. After being received and sorted, eggs were put in cold storage, where they were fogged daily using a 10% concentration of a proprietary disinfectant (Viroshield, Kilco) containing glutaraldehyde and benzalkonium chloride. They were then incubated for around 18 days in single-stage setters before being transferred into hatcher baskets by machine. These were placed in hatcher incubators in several hatcher rooms and formaldehyde evaporation was employed within hatchers as an antimicrobial measure during hatching. Hatched chicks were manually removed after three to four days and sorted in the chick handling area where, depending on requirements, chicks were sexed, vaccinated and transferred into transport crates for delivery. A macerator adjacent to the egg handling area piped egg waste and hatcher debris into sealed outdoor skips. Used trolleys, egg trays, hatcher baskets and chick delivery baskets were cleaned in separate wash machines.

### 2.2. Advice and Selection of Disinfectants

A written report with advice on *Salmonella* control followed each hatchery visit by the Animal and Plant Health Agency (APHA) team. These reports reflected observations made during the visit, the visit results and any changes to management that had been made between visits. The expert advice focused mainly on C&D procedures for equipment and washers, but other potential control measures were also highlighted. Advice was tailored and was responsive to information and observations acquired at the visit, as well as microbiological results, rather than being standardised by intervention protocol or operating procedure.

### 2.3. Sampling

**Intensive sampling visits.** There were seven intensive sampling visits between May 2015 and August 2019. Intervals between visits varied from two months to over a year, depending on hatchery events and research funding. Sampling areas included egg reception, sorting and cold storage areas, setter area, egg transfer area, hatchers, chick handling area, tray wash areas, waste processing areas and delivery lorries. Between 351 and 394 samples were collected each time, according to a written protocol that dictated a sequence following the flow of eggs and chicks through the hatchery. The protocol specified what elements to sample with further detail where needed, for example, specifying the removal of covers before sampling drains. Target numbers and locations of swabs were given for key elements such as setters and hatchers, plus all drains present, although some discretion was left to account for changing patterns of usage and ease of access. Specified elements to sample were: floors, walls, doors, drains, trolleys, trays, setters, hatchers (including ventilator extraction corridors) and equipment including for cleaning and personal protection. Floors and drains in access corridors and lobbies of setter and hatcher rooms were explicitly specified. Further details of sampling sites are provided in the Supplementary Data (Table S1).

Additionally, sampling focused on areas where contamination was found during the previous visit. Sampling followed an established protocol, previously used in hatcheries, for sensitive detection of motile *Salmonella* from environmental sources [14]. Using a clean disposable vinyl glove each time, an area of approximately 0.5 m^2^ was sampled with a large gauze swab (900 cm^2^ Readiwipes Dry: Robinson Healthcare Ltd., Worksop, UK) that had been autoclaved in 225 mL of buffered peptone water (BPW, Merck: Merck Millipore, Watford, UK). The swab was replaced in the jar of BPW and returned to the APHA Weybridge laboratory at ambient temperature for culture on the same day.

**Special visit.** This visit, in March 2018, was to assess the effectiveness of C&D and to compare *Salmonella* detection from samples collected into differing volumes of BPW. Consequently, it did not follow the all-area sampling approach otherwise adopted. Samples were collected from chick handling areas (40) and waste areas (20), before and after C&D. Sampling was also conducted in duplicate from adjacent 0.5 m^2^ surfaces in chick handling areas (40 pairs) and from waste and outside areas (40 pairs). One each of these paired swabs came from, and was replaced into, the usual (225 mL) quantity of BPW, whilst the other was moistened with and replaced into only 30 mL of BPW.

**Additional samples taken by broiler company management.** Sponge swabs from all areas of the hatchery and fluff samples from hatchers were collected by hatchery staff and posted at ambient temperature to APHA Weybridge between 2015 and 2019. These samples were used to monitor antimicrobial resistance amongst *Salmonella* isolates.

**Breeding farms.** All 60 breeding flocks supplying eggs to the hatchery during this study were repeatedly sampled on-farm by company field managers between October 2017 and March 2018. For each flock, five pairs of boot swabs (pre-moistened swabs slipped over clean plastic overshoes) were collected, each time using at least 100 shuffled steps over a representative part of the poultry house floor. Dust samples from ledges and other accumulation points across the houses were collected into clean plastic bags using clean disposable gloves. Samples were posted to APHA Weybridge.

### 2.4. Bacteriological Procedures

Postal samples were placed in 225 mL BPW on receipt and samples transported in 30 mL BPW had 200 mL BPW added. All samples were processed by a modified version of ISO 6579:2017, using pre-enrichment in BPW followed by culture on modified semi-solid Rappaport-Vassiliadis agar and Rambach agar, as previously described [14]. Slide agglutination tests were performed, using polyvalent somatic and flagella antisera, to confirm *Salmonella* from representative suspect colonies. All positive isolates were serotyped according to the White–Kaufmann–Le Minor Scheme [41].

*S.* 13,23:i:- isolates from the postal hatchery samples (n = 643) were subjected to disc diffusion tests using ISO-Sensitest agar (Oxoid; Oxoid Ltd., Basingstoke, UK) and discs (Oxoid) containing the following antimicrobials: amikacin 30 µg, amoxycillin/clavulanic acid 30 µg, ampicillin 10 µg, apramycin 15 µg, ceftazidime 30 µg, cefotaxime 30 µg, chloramphenicol 30 µg, ciprofloxacin 1 µg, furazolidone 15 µg, gentamicin 10 µg, nalidixic acid 30 µg, neomycin 10 µg, streptomycin 10 µg, trimethoprim/sulphamethoxazole (1:19) 25 µg, compound sulphonamides 300 µg and tetracycline 10 µg. British Society for Antimicrobial Chemotherapy (BSAC) breakpoints were used. If there was no BSAC breakpoint available, then published breakpoints were used [42].

### 2.5. Statistical Analysis

Data were assigned to seven broad sample categories: egg area, setter area, hatcher area, chick handling area, tray wash area, waste/outside area and miscellaneous. Analysis by logistic regression examined findings from the different visits separately, identifying which visits and sample categories had the highest odds of yielding positive samples. The model was as follows:

$$logit\left(p_{i,j}\right)=log\left(\frac{p_{i,j}}{1-p_{i,j}}\right)=\alpha_{i}+\beta_{j}+e$$

where $p_{i,j}$ is the proportion of positive samples, parameter $\alpha_{i}$ is the relative coefficient for sample category *i*, $\beta_{j}$ is the farm visit *j* and e is a normally distributed error term with mean zero. The equation above can be solved for *p_i,j_* to find the proportion of positive samples, assuming that the effects of sample category and visit do not interact. To assess the goodness-of-fit of the model, McFadden’s pseudo-R2 value and Hosmer–Lemeshow goodness-of-fit test were used [43,44].

To compare the two swab methods (in 30 mL or 225 mL of BPW), McNemar’s two-sample paired proportion test [45] was used to determine whether there was a significant difference in the outcomes from the two methods. The Wilson Score Interval [46] was used to estimate the 95% confidence intervals for sensitivity.

All analyses were performed in Stata 15 [47].

## 3. Results

### 3.1. Hatchery Visits

**Figure 1:**
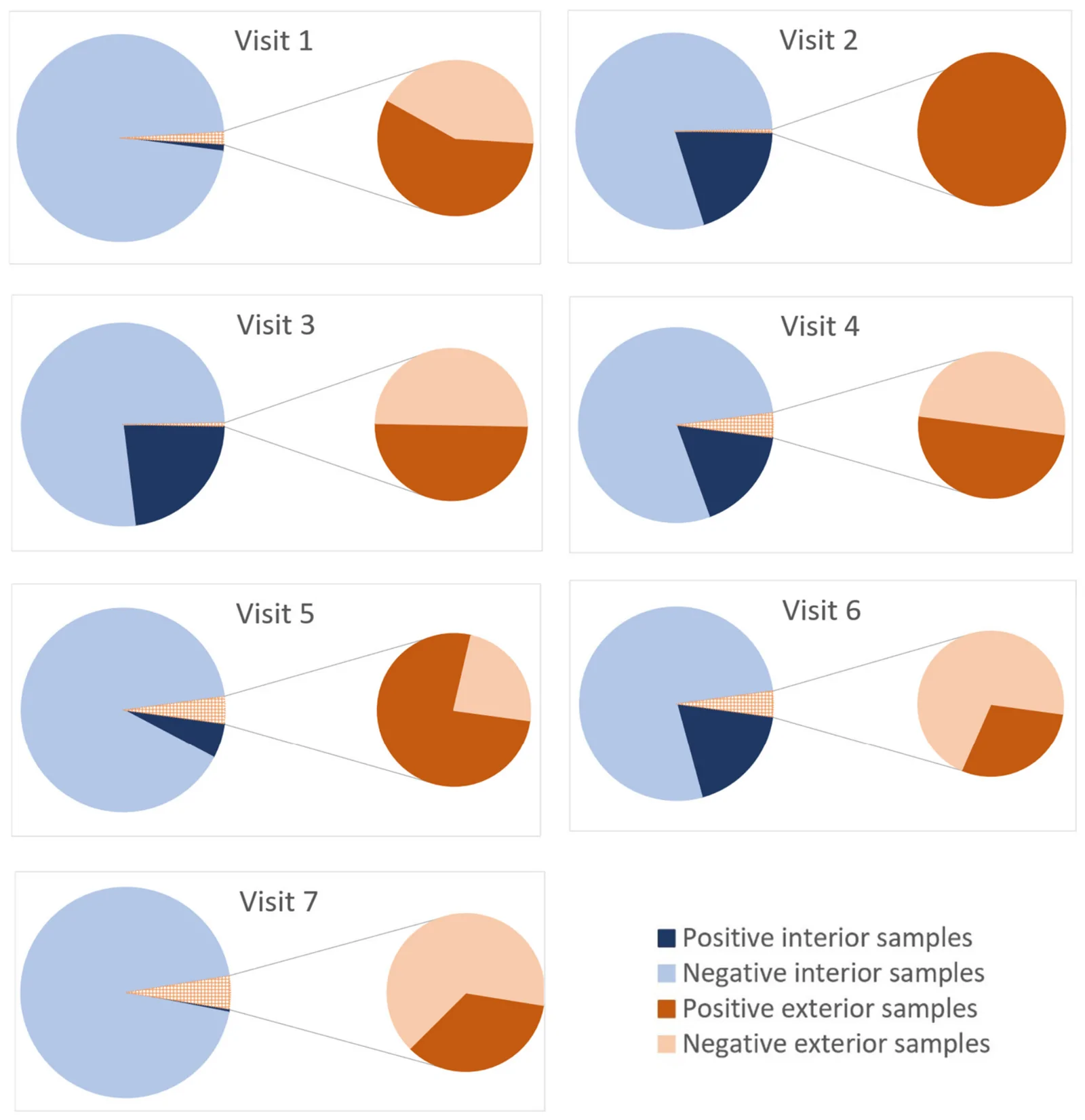
The proportions of samples collected from the interior and exterior parts of the hatchery that were *Salmonella*-positive at each of the seven hatchery visits.

**Longitudinal sampling.** Of the 2645 samples collected over the seven visits, 361 (13.6%) yielded *Salmonella*. Between 2.2% and 23.1% of the samples per visit were *Salmonella*-positive. The findings are illustrated in Figure 1, with the detailed results shown in Table 1. There was a substantial variation over time in the degree of *Salmonella* contamination for some areas. For example, the waste and external areas were contaminated at all visits, whereas the egg and setter areas yielded no *Salmonella* on three visits (1, 5 and 7) when the hatchery was less contaminated in general.

The egg handling areas consistently had the lowest contamination (2.1% of samples positive overall), with no isolations on four occasions. There was progressively greater contamination along the production process: setters (3.6% overall), hatchers (19% overall) and chick handling areas (28% overall). The combined ‘waste and outside’ areas showed the heaviest contamination (58%) of any specific area. At all the visits except for the final one, there was a high proportion of positive samples from the macerator and its surroundings.

The various C&D regimes employed during this study are described below and summarised in Table 2. The first visit (May 2015) followed an intensive clean of the hatchery, with the in-house sampling still yielding sporadic isolations of *S.* 13,23:i:- from drains. The disinfection regime was long-standing, using disinfectants based on the quaternary ammonium compound (QAC) benzalkonium chloride (BAC) and, intermittently, the oxidizing agent peroxymonosulphate. Both of these disinfectants were being applied at concentrations lower than specified both by the UK Department for Environment, Food and Rural Affairs (i.e., Defra General Orders concentrations, where the test organism is *Salmonella* Enteritidis) and by their manufacturers. The visit yielded S. 13,23:i:- from eight samples: swabs from a drain and a cleaning tool (the latter classified under Miscellaneous) in the tray wash room and six from the waste areas (macerator plus an external concrete apron accommodating the waste skip).

Visit 2 was over two years later, in August 2017. Only the BAC-based disinfectant was then in use because of concerns about corrosion from peroxymonosulphate. The BAC-based disinfectant was being used at a low concentration in order to counter the issues of blocked power wash jets and slippery floor surfaces associated with a full-concentration application. *S.* 13,23:i:- was isolated from all areas, with an overall proportion of 20% positive samples and with the hatcher basket wash (50%) and waste/outside areas (89%) being most heavily contaminated. Subsequently, at Visits 3 (October 2017) and 4 (January 2018), a glutaraldehyde-plus-BAC compound product had replaced the low-concentration BAC disinfectant. The proportion of samples positive for *S.* 13,23:i:- remained high, around 20%, although locally, the proportions fell substantially between these visits in both the chick handling (77% vs. 34%) and the tray wash (67% vs. 15%) areas.

Visit 5 (July 2018) followed the C&D assessment in March 2018 and a major upgrade of the C&D program, which used a compound disinfectant (glutaraldehyde plus the QAC didecyldimethylammonium chloride) and the additional intermittent application of a peroxymonosulphate disinfectant followed by rinsing. The overall proportion of *Salmonella*-positive samples had approximately halved to 9%, there were no isolations from the egg or setter areas, and contamination was modest in the hatcher and chick handling areas. However, over a quarter of the tray wash area samples were positive, including some taken from the ‘clean’ section and from the water reservoir of the hatcher basket wash machine. The waste skip and the lorry wash areas remained heavily contaminated.

Visit 6 (November 2018) followed a further deep clean, involving some equipment (including the macerator) being dismantled. There was a resurgence in *S.* 13,23:i:- throughout the hatchery, with the hatchers and the chick handling areas being notably affected (30% and 60% of samples positive, respectively). All the samples from within and around the macerator were also positive, and there was frequent *Salmonella* contamination of the floor in the hatcher basket store. 

The final visit in August 2019 followed the implementation of a higher peroxymonosulphate disinfectant concentration followed by rinsing. The hatcher basket washer and the loft space above it had also been deep cleaned. The indoor *Salmonella* contamination had dramatically reduced, with *S.* 13,23:i:- being isolated once, from the hatcher debris that had accumulated around the chick take-off line, and *S.* Idikan being recovered from a pair of wellington boots sampled indoors but used outside. *S.* 13,23:i:- and *S.* Idikan were isolated from external areas, including puddles around the skip and the lorry parking area and the new waste tank.

A large majority of *Salmonella* isolates over all the visits (357 of 361) were *S.* 13,23:i:-. Only two other serovars were identified: *S.* Give, found once during the first visit on a waste skip outdoors, and *S.* Idikan during Visit 7 from boots and outdoor puddles.

The logistic regression model (Table 3) suggests a significantly increased risk of *Salmonella* isolation in the samples from the waste, chick handling, post-hatch cleaning (tray wash) and hatcher areas compared to the setter areas (all *p* < 0.001). The waste area showed the greatest increase in risk with an odds ratio (OR) of 93.0 (95% CI 51.1–169.2) compared to the setter areas. The egg and miscellaneous areas did not show a significantly different risk compared to the setter areas. All the later visits, apart from the seventh, showed a significantly increased risk of positive *Salmonella* samples compared to Visit 1, but no clear pattern was observed in terms of the change in risk over time. Visit 7 showed a non-significant reduced risk of *Salmonella* isolation compared to Visit 1, with an OR of 0.600 (95% CI 0.219–1.644). If the same model is run with Visit 7 as the baseline category (Supplementary Data; Table S2), it is observed that this visit had a significantly lower risk of *Salmonella* isolation compared to each of Visits 2 through 6 (all *p* < 0.001).

The pseudo-R2 of 29.1% is low, which is to be expected as this model only included two factors (visit and location), and the Hosmer–Lemeshow goodness-of-fit statistic suggests the model is incomplete (*p* = 0.002).

**Effectiveness of cleaning and disinfection.** Before C&D, 22% of the samples yielded *S.* 13,23:i:-, with the waste area samples being more often positive (9/20) than the chick handling area (4/40). After C&D, only one of the 60 (1.7%) samples yielded *Salmonella*.

**Comparison of swabs transported in different volumes of buffered peptone water.** There was a similar frequency of *Salmonella* recovery from the paired sites with swabs transported in either 30 mL or 225 mL BPW (Table 4). For the 30 mL samples, 39 of 80 samples were positive, versus 35 of 80 for the 225 mL samples, and there were discrepant negative results for one of 40 and five of 40 for the 30 mL and 225 mL volumes, respectively. McNemar’s test of a difference between the two methods was not significant (*p* = 0.103). Assuming the 40 sites that did not yield *Salmonella* with either method were genuinely *Salmonella*-negative, the corresponding sensitivities of detection were 97.5% (95% CI 87.1–99.6%) for the 30 mL samples and 87.5% (95% CI 73.9–94.5%) for the 225 mL samples. Overall, there was a high consensus between the two BPW transport volumes, with 74/80 samples (92.5%, 95% CI 84.6–96.5%) showing agreement.

### 3.2. Antimicrobial Resistance

Most (96%) of the 643 *S.* 13,23:i:- postal sample isolates were fully sensitive to all the agents in the panel (Table 5). This proportion varied by year, with the lowest (79%) being in 2017. However, only 34 isolates were tested that year. Nalidixic acid (nal) was the only compound to which resistance was documented in all years. Other resistances showed much more variable occurrence, and neither multi-resistance (resistance to three or more classes of antimicrobials) nor resistance to extended-spectrum cephalosporin drugs was observed.

### 3.3. Breeding Flock Samples

None of the samples (476 sets of boot swabs and 598 dust samples) from the 60 broiler parent breeding flocks yielded *Salmonella*.

### 3.4. Advice and Changes to Management

The key advice given was related to C&D and to the operation of the tray washer. The changes to the disinfectants are detailed in the visit results and in Table 2. Other specific advice, following the persistent and frequent isolation of *Salmonella* from an intended decontamination area (tray washer), was to increase the washer working temperature to above 60 °C, to increase the routine concentration of disinfectant within the machine, to incorporate an acidic wash of the machine at the end of the day, and to avoid recontamination of the washed hatcher baskets by dust from the overhead loft space.

## 4. Discussion

*Salmonella* control is a challenge for hatcheries in the UK and elsewhere [10,14,22,48,49,50]. Hatcheries are a potential barrier between the layers of the production pyramid for microbes, but they are also risk sites for the cross-contamination and dissemination of pathogens [12,28]. In the UK and elsewhere, the consolidation of the poultry industry has led to fewer and larger hatcheries, each supplying increased numbers of production flocks [13]. The existing mandatory monitoring of broiler, layer and breeder birds and chick box liner papers for *Salmonella* (under National Control Programmes in the EU and UK) should quickly allow hatcheries producing chicks carrying regulated serovars to be identified. In the event that this happens, there may be substantial consequent economic and, potentially, supply effects for the industry [51]. Investing in the effective control of all *salmonellae* in hatcheries reduces this risk, thereby protecting the food chain from all Salmonella serovars.

In the present case, *Salmonella* was recovered during every visit to a large commercial hatchery. Multifocal *Salmonella* contamination was revealed during the initial sampling, which was triggered by the isolation of a *Salmonella* serovar of relevance to public health under routine surveillance (which was not found again), and a longitudinal study of C&D was pursued. Nearly all the isolates were *S.* 13,23:i:-, and the low contamination within the egg handling areas [22], plus no isolation of *S.* 13,23:i:- after sensitive sampling in the environments of all the flocks supplying the eggs, indicated that *S.* 13,23:i:- was very likely a resident rather than a strain that had been recently or repeatedly introduced through contaminated eggs. Two recently surveyed UK hatcheries have also had *S.* 13,23:i:- isolated during multiple visits, suggesting resident contamination, although some flocks supplying these hatcheries had yielded *S.* 13,23:i:- [14]. For the present hatchery, the original route for introduction of *S.* 13,23:i:- is unknown.

As the present work had an interventional aspect, was subject to variation in access according to usage of the setters and the hatchers and was not simply a longitudinal survey, repeat sampling was not performed to a strict template. The samples were focused on perceived problem areas and the sampling was enhanced in some areas from visit to visit, this being informed by the earlier results. Therefore, whilst the proportion of positive samples in an area at any one visit may be regarded as being broadly indicative of the intensity of contamination, there was sampling bias and less can be read into smaller and fluctuating differences over time or between locations. Furthermore, in the absence of extensive molecular genetic characterisation of the isolates, *Salmonella* clones could not be tracked through the premises. Nonetheless, some consistent and logical patterns were observed.

Surface and equipment contamination increased along the hatchery process pathway, with the lowest proportion of isolations from the egg areas and the setters and the chick handling areas being the most heavily contaminated internal areas. The logistic model gives an indication of the possible comparative range of *Salmonella* risk in certain areas of the hatchery. However, it should not be considered definitive or generalisable on its own. This is a case study at a single hatchery and, with only two factors, the model is clearly incomplete. The goodness-of-fit statistics confirm that it is a limited model, only explaining 29% of the variation. There may be many other variables not measured in this case study that could affect the likelihood of a particular sample being positive for *Salmonella*. Nonetheless, previous studies have consistently found a similar pattern of contamination, with the hatchers and the hatchling handling areas often being heavily contaminated [5,7,10,14,22,23,25,26]. This reflects the generation of dust and other contaminants during hatching [29] and its further spread during the processing and handling of the chicks. 

During some visits, the hatcher interiors were heavily contaminated despite recent C&D. Hatchers are difficult to clean and disinfect, as chick fluff and dust can be hidden within the air vents, the ducting for the belts that drive the fans, and the plenum corridors [7,10,21,22,26]. During earlier visits (2, 3 and 4), the air vents in the plenum corridors were contaminated. If these ancillary areas are missed during C&D, then the negative pressure and turbulence when restarting the ventilation systems may draw dust in from such areas, which can then cause recontamination even after an apparently effective hatcher C&D [21].

The tray wash areas are also often contaminated with *Salmonella* [7,22,25,26]. Poorly effective C&D of hatcher baskets and transport crates increases the risk of *Salmonella* spread through the production chain [7,22,52,53]. There was evidence of resident contamination of the hatcher basket wash machine in this hatchery, as previously reported elsewhere [14]. Following the advised changes in operation and the focused decontamination of the overhead loft area, considerable improvement was observed, with no *Salmonella* isolated from this area during the final visit.

The waste and exterior areas showed the heaviest contamination during all the visits except one. The macerator was a particular focus of indoors contamination except during the final visit. The waste processing areas were highly contaminated in other UK chicken [14] and duck [25] hatcheries, and they are often close to the hatcher basket washing and storage areas. Most of the samples from the external areas yielded *S.* 13,23:i:-, and the samples from the skips, the surrounding areas and the puddles around the lorry loading areas were heavily contaminated. These areas are less likely to experience regular C&D, thus posing a potential biosecurity vulnerability for both the export and the import of contamination. As an illustration, *S.* Idikan was isolated from boots kept on the premises that were stored indoors but used outside. There was also other *Salmonella*-contaminated mobile equipment, such as cleaning tools, highlighting the need to retain such items within designated areas and to keep them effectively disinfected. 

Exterior hatchery contamination poses a biosecurity risk to rearing and breeding farms via vehicles and drivers. Indeed, a second hatchery in the same company yielded *S.* 13,23:i:- from a swab taken around a waste tank. Whole genome sequencing indicated a high degree of relatedness of four to six core genome single nucleotide polymorphisms (SNPs) between this isolate and those from the study hatchery (unpublished data). Although not conclusive evidence for transfer, this illustrates the risk posed by vehicles, waste containers and personnel moving between such contaminated locations.

Given that one key target of C&D in hatcheries is the control of contamination by *Salmonella* spp., variations during the study period in C&D (i.e., methods, focus and disinfectants) probably influenced the longitudinal variation in the hatchery’s *Salmonella* contamination. However, other potential factors, including general management and environmental conditions, were not controlled for. Historically, C&D relied on a BAC-based disinfectant at low concentrations plus regular peroxymonosulphate disinfectant application. This substantially reduced, but did not eliminate, the *S.* 13,23:i:- contamination. Furthermore, formaldehyde evaporation during hatching did not prevent hatcher contamination, and residual organic matter and biofilm was observed after cleaning. It was suspected that *S.* 13,23:i:- biofilms were likely to be hindering disinfection [54,55,56,57].

The dramatic increase in contamination observed at the second visit followed the cessation of peroxymonosulphate disinfection (after concerns about corrosion and staff safety), leading to a heavy reliance on a low-concentration BAC-based disinfectant. There had also been a change in management personnel during the two years since the first visit. Contamination remained high at the third visit, despite the hatchery having introduced a glutaraldehyde/BAC disinfectant product. Following an additional visit assessing C&D, a mix of glutaraldehyde-plus-QAC (non-BAC) and oxidizing disinfectants was advised to more effectively address biofilms [58] and, by the rotation of agents, to counter the development of biocide tolerance [59,60,61,62].

However, the *Salmonella* contamination remained intractable. Dismantling equipment, including the macerator, to facilitate deep C&D may have dislodged dust that contained *S.* 13,23:i:-, leading to transiently higher contamination during Visit 6. Nonetheless, major improvements to the protocol and the standard of C&D, with a particular focus on the hatcher basket wash facility, culminated in a dramatic reduction in isolation frequency (from 20% to 2.3% of samples) between the sixth and seventh visits.

During the final (seventh) visit, only 0.5% (2/373) of the samples from inside the hatchery yielded *Salmonella*, and only one of these was *S.* 13,23:i:-. This came from the waste in the chick take-off area, indicating a much-reduced resident *Salmonella* and a lowered risk of infection in hatched chicks. The core genomes of the *S.* Idikan and *S.* 13,23:i:- isolates from the exterior areas during the final visit were all within a 10 SNP cluster (unpublished data), suggesting that the *S.* Idikan isolates were derived from resident *S.* 13,23:i:- strains that had regained the ability to express phase two flagellar antigens. 

Resistance to the tested antimicrobial agents was infrequent among the *S.* 13,23:i:- isolates collected by the hatchery staff over the course of the study, and did not show a strong pattern over more than four years. Only nal resistance was seen every year. A chromosomal topoisomerase mutation is usually responsible for nal resistance in salmonellae, which often also reduces susceptibility to the medically important fluoroquinolone antimicrobials [63,64]. Indeed, there was a single isolate in 2017 that was resistant to both nal and ciprofloxacin. Nonetheless, the isolates in the present study appeared to be more commonly fully susceptible than did contemporaneous *Salmonella* reported in aggregated data from British poultry [65]. The infrequent, varying resistances among the serial *S.* 13,23:i:- isolates without antibiotic selection pressures suggests some genetic diversity, possibly influenced by disinfectant pressures and/or the occasional introduction of new strains. Some disinfectants, especially BAC used at low concentrations, can drive both biocide and antimicrobial resistance [66,67,68]. It is prudent to monitor resistance in hatchery microbiota, as studies have shown the transmission of resistant hatchery contaminants to farms [69,70]. 

The present finding that a 30 mL BPW transport volume for hatchery swabs might yield more sensitive *Salmonella* detection than 225 mL is supported by similar findings from sampling feed mills (APHA unpublished data). A consistent difference, if it can be confirmed in an independent prospective study, may reflect a lower risk of overgrowth of competing bacteria in the smaller volume before incubation. Such overgrowth may occur when there is an extended time before incubation during long sampling investigations, and/or when ambient temperatures are warm. Therefore, 30 mL BPW swab samples may sometimes provide a more stable sample option, whilst also being less bulky during field investigations. 

It was not possible to eliminate the established *S.* 13,23:i:- during the present study, although when the intensive sampling ended there was a low frequency of *Salmonella* recovery, and this was exclusively from the waste and external areas. The current work illustrates some particular challenges with environmental *Salmonella* control in hatcheries. These include the application of some disinfectants at concentrations below those used on farms (typically in order to protect equipment from corrosion and to reduce chemical hazards to staff) and the presence of much equipment with parts that are poorly accessible for C&D. Such equipment also has the potential for the liberation of trapped *Salmonella*-bearing debris if the equipment is dismantled for decontamination. Further hatchery *Salmonella* risks include breaches of external biosecurity via frequent egg deliveries and vehicle movements and the constant cycling of equipment around the hatchers and the chick handling areas.

The practical application and performance of disinfectants in specific field situations is subject to many influences that may not be accounted for by laboratory-based licensing tests [71]. Furthermore, some hatchery strains of *Salmonella* may develop tolerance to QAC-based disinfectants [55]. Therefore, an efficacious protocol may only be arrived at after a multi-stage approach involving field monitoring and attention to cleaning as well as disinfection [72]. The present study offers useful insights in this regard, with evidence that repeated and comprehensive sampling can help to identify the existence and possible causes of inadequate C&D, as well as highlighting localised contamination issues. However, the interpretation of cause and effect in the complex environment of a hatchery is limited in the present case. Following from this, it cannot be known with confidence how generalisable are the findings of this study from a single site. Furthermore, although these investigations did not find evidence of incoming contamination from eggs, in the absence of high-resolution typing of isolates the possibility that external re-contamination was a factor in the observed patterns of contamination cannot be entirely discounted.

There are certain practices that are key factors for hatchery *Salmonella* control, in addition to identifying and attending to ‘problem areas’. These include strict management to ensure that high biosecurity is maintained, alongside C&D standards sufficient to remove residual organic matter and limit biofilm formation. This approach was demonstrated in the present case, where a low *Salmonella* risk from incoming eggs and awareness of exterior contamination was combined with stepwise enhancements in the disinfection regime, in addition to focused C&D interventions guided by repeated sampling throughout the premises.

## Figures and Tables

**Table 1:** *Salmonella* isolations from the hatchery at each visit.

Visit (Months) ^a^	Number of *Salmonella*-Positive Samples/Number of Samples							
	Egg Handling	Setters	Hatchers ^b^	Chick Handling ^b^	Tray Wash ^b^	Waste/Outside ^b^	Misc.	Totals by Visit
1	0/53	0/119	0/99	0/29	1/43	1 ^d^ + 5/8	1/11	8/362 *(2.2%)*
2 ^c^ (+27)	3/56	11/142	33/98	12/33	4/8	8/9	3/16	74/362 *(20.4%)*
3 ^c^ (+2)	3/56	7/142	35/91	24/31	4/6	7/9	1/16	81/351 *(23.1%)*
4 ^c^ (+3)	1/46	13/135	30/100	13/38	6/39	13/23	0/9	76/390 *(19.5%)*
5 ^c^ (+6)	0/38	0/126	3/102	2/42	14/53	16/22	0/11	35/394 *(8.9%)*
6 ^c^ (+4)	0/38	2/126	30/99	25/42	9/56	12/22	0/9	78/392 *(19.9%)*
7 (+9)	0/44	0/121	0/97	0/52	0/45	2 ^e^ + 6/28	1 ^e^/7	9/394 *(2.3%)*
**Totals by area**	7/331 (*2.1%*)	33/911 (*3.6%*)	131/686 (*19.1%*)	76/267 (*28.5%*)	38/250 (*15.2%*)	70/121 (*57.9%*)	6/79 (*7.6%*)	

^a^ Time (months) since previous visit. ^b^ Areas with significantly (*p* < 0.001) higher likelihood of *Salmonella*-positive samples compared with setters. ^c^ Visits with significantly (*p* < 0.001) higher likelihood of *Salmonella*-positive samples, compared with Visit 1. ^d^ *Salmonella* Give, ^e^ *Salmonella* Idikan. All other isolates were *Salmonella* 13,23:i:-.

**Table 2:** Summary of cleaning and disinfection regimes during the present study.

Visit No.	Visit Date	Biocides ^a^ in Use	Notes
1	May 2015	BAC (quaternary ammonium) routinely. PMS (oxidising) periodically.	Recent deep-clean. Both BAC and PMS used at below-recommended concentrations.
2	Aug. 2017	BAC	BAC used at below-recommended concentration. PMS stopped because of corrosion issues.
3	Oct. 2017	GLD (aldehyde) + BAC routinely	
4	Jan. 2018	GLD + BAC routinely	
-	Mar. 2018	GLD + BAC routinely	Cleaning and disinfection study visit. Not a full sampling visit.
5	July 2018	GLD + DDAC (quaternary ammonium) routinely. PMS periodically.	Rinsing after PMS use.
6	Nov. 2018	GLD + DDAC routinely. PMS periodically.	Following deep-clean of equipment including macerator. Rinsing after PMS use.
7	Aug. 2019	GLD + DDAC routinely. PMS periodically.	Following deep-clean of hatcher basket and loft space above. PMS concentration increased, rinsing after use.

^a^ Principal agents in the commercial disinfectants employed: BAC—benzalkonium chloride, PMS—peroxymonosulphate, GLD—glutaraldehyde, DDAC—didecyldimethylammonium chloride.

**Table 3:** Multivariable logistic regression analysis of factors ‘Location category’ and ‘Visit number’ associated with the odds of a sample returning a positive *Salmonella* result.

Variable	Odds Ratio	95% Confidence Interval	*p*-Value
**Location category**			
Setters	Reference		
Chick Handling	15.497	(9.723–24.699)	<0.001
Tray Wash	8.694	(5.130–14.736)	<0.001
Egg Handling	0.562	(0.245–1.291)	0.175
Hatchers	7.662	(5.093–11.527)	<0.001
Miscellaneous	1.988	(0.792–4.991)	0.143
Waste/Outside	93.021	(51.149–169.172)	<0.001
**Visit number**			
1	Reference		
2	19.076	(8.656–42.040)	<0.001
3	24.477	(11.104–53.958)	<0.001
4	12.039	(5.505–26.328)	<0.001
5	3.548	(1.563–8.052)	0.002
6	11.418	(5.234–24.907)	<0.001
7	0.600	(0.219–1.644)	0.321

**Table 4:** Classification of 80 samples as positive or negative for *Salmonella* in 30 mL and 225 mL volumes of BPW.

		**225 mL**		**Total** **(30 mL)**
		Positive	Negative	
**30 mL**	Positive	34	5	39
	Negative	1	40	41
**Total (225 mL)**		35	45	80

**Table 5:** Numbers of antimicrobial resistant isolates of *Salmonella* 13,23:i:- from postal samples.

Antimicrobial Resistance Pattern	Year (Number of Isolates Tested)				
	2015 (75)	2016 (43)	2017 (34)	2018 (361)	2019 (130)
Fully susceptible	72	42	27	348	129
AM				1	
NA		1	5	2	1
S	2				
SU				4	
T				1	
FR, T				1	
NA, T	1				
S, SU				2	
SXT, T				1	
C, CIP, NA			2		
SU, SXT, T				1	

AM—ampicillin, C—chloramphenicol, CIP—ciprofloxacin, FR—furazolidone, NA—nalidixic acid, S—streptomycin, SU—sulphonamide compounds, SXT—sulphamethoxazole and trimethoprim, T—tetracycline.

## Data Availability

The original contributions presented in this study are included in the article and the Supplementary Materials. Further inquiries can be directed to the corresponding author.

## Supplementary Materials

The following supporting information can be downloaded at: https://www.mdpi.com/article/10.3390/microorganisms14081747/s1, Table S1: Details of sampling locations and results; Table S2: Multivariable logistic regression analysis of factors ‘Location’ and ‘Visit number’ associated with the odds of a sample returning a positive *Salmonella* result.

## References

[1] EFSA, ECDC (2024). The European Union One Health 2023 Zoonoses report. EFSA J..

[2] Bryan F.L., Doyle M.P. (1995). Health risks and consequences of *Salmonella* and *Campylobacter jejuni* in raw poultry. J. Food Prot..

[3] Wang J., Vaddu S., Bhumanapalli S., Mishra A., Applegate T., Singh M., Thippareddi H. (2023). A systematic review and meta-analysis of the sources of *Salmonella* in poultry production (pre-harvest) and their relative contributions to the microbial risk of poultry meat. Poult. Sci..

[4] Pires S.M., Vieira A.R., Hald T., Cole D. (2014). Source attribution of human salmonellosis: An overview of methods and estimates. Foodborne Pathog. Dis..

[5] Kim A., Lee Y.J., Kang M.S., Kwag S.I., Cho J.K. (2007). Dissemination and tracking of *Salmonella* spp. in integrated broiler operation. J. Vet. Sci..

[6] Gosling R., Oastler C., Nichols C., Jackson G., Wales A.D., Davies R.H. (2022). Investigations into *Salmonella* contamination in feed mills producing rations for the broiler industry in Great Britain. Vet. Sci..

[7] Davies R., Breslin M., Corry J.E., Hudson W., Allen V.M. (2001). Observations on the distribution and control of *Salmonella* species in two integrated broiler companies. Vet. Rec..

[8] Wierup M., Wahlstrom H., Lahti E., Eriksson H., Jansson D.S., Odelros A., Ernholm L. (2017). Occurrence of *Salmonella* spp.: A comparison between indoor and outdoor housing of broilers and laying hens. Acta Vet. Scand..

[9] Davies R., Liebana E., Breslin M. (2003). Investigation of the distribution and control of *Salmonella enterica* serovar Enteritidis PT6 in layer breeding and egg production. Avian Pathol..

[10] Davies R.H., Nicholas R.A., McLaren I.M., Corkish J.D., Lanning D.G., Wray C. (1997). Bacteriological and serological investigation of persistent *Salmonella enteritidis* infection in an integrated poultry organisation. Vet. Microbiol..

[11] Volkova V.V., Bailey R.H., Wills R.W. (2009). *Salmonella* in broiler litter and properties of soil at farm location. PLoS ONE.

[12] Wales A., Davies R. (2020). Review of hatchery transmission of bacteria with focus on *Salmonella*, chick pathogens and antimicrobial resistance. Worlds Poult. Sci. J..

[13] Withenshaw S.M., Cawthraw S., Gosling B., Newton K., Oastler C.E., Smith R.P., Davies R.H. (2021). Risk factor analysis for *Salmonella* contamination of broiler chicken (*Gallus gallus*) hatcheries in Great Britain. Prev. Vet. Med..

[14] Oastler C.E., Nichols C., Newton K., Cawthraw S., Gosling R.J., Martelli F., Wales A.D., Davies R.H. (2022). Observations on the distribution and control of *Salmonella* in commercial broiler hatcheries in Great Britain. Zoonoses Public Health.

[15] Bailey J.S., Stern N.J., Fedorka-Cray P., Craven S.E., Cox N.A., Cosby D.E., Ladely S., Musgrove M.T. (2001). Sources and movement of *Salmonella* through integrated poultry operations: A multistate epidemiological investigation. J. Food Prot..

[16] Racicot M., Leroux A., Ng S., Comeau G., Quessy S.G., Gaucher M.-L. The Canadian food inspection agency establishment-based risk assessment model for hatcheries: Principles and algorithm. Proceedings of the IAFP European Symposium on Food Safety.

[17] EFSA (2009). Quantitative estimation of the impact of setting a new target for the reduction of *Salmonella* in breeding hens of *Gallus gallus*. EFSA J..

[18] Koutsoumanis K., Allende A., Alvarez-Ordóñez A., Bolton D., Bover-Cid S., Chemaly M., De Cesare A., Herman L., EFSA, BIOHAZ (2019). *Salmonella* control in poultry flocks and its public health impact. EFSA J..

[19] Sivaramalingam T., McEwen S.A., Pearl D.L., Ojkic D., Guerin M.T. (2013). A temporal study of *Salmonella* serovars from environmental samples from poultry breeder flocks in Ontario between 1998 and 2008. Can. J. Vet. Res..

[20] McMullin P.F. (2009). Hygiene and microbiological control in hatcheries. Avian Biol. Res..

[21] Christensen J.P., Brown D.J., Madsen M., Olsen J.E., Bisgaard M. (1997). Hatchery-borne *Salmonella enterica* serovar Tennessee infections in broilers. Avian Pathol..

[22] Davies R.H., Breslin M.F. (2004). Observations on the distribution and control of *Salmonella* contamination in poultry hatcheries. Br. Poult. Sci..

[23] Cox N.A., Bailey J.S., Mauldin J.M., Blankenship L.C. (1990). Presence and impact of *Salmonella* contamination in commercial broiler hatcheries. Poult. Sci..

[24] Kalily E., Hollander A., Korin B., Cymerman I., Yaron S. (2017). Adaptation of *Salmonella enterica* serovar Senftenberg to linalool and its association with antibiotic resistance and environmental persistence. Appl. Environ. Microbiol..

[25] Martelli F., Birch C., Davies R.H. (2016). Observations on the distribution and control of *Salmonella* in commercial duck hatcheries in the UK. Avian Pathol..

[26] Mueller-Doblies D., Clouting C., Davies R.H. (2013). Investigations of the distribution and persistence of *Salmonella* and ciprofloxacin-resistant *Escherichia coli* in turkey hatcheries in the UK. Zoonoses Public Health.

[27] Pedersen T.B., Olsen J.E., Bisgaard M. (2008). Persistence of *Salmonella* Senftenberg in poultry production environments and investigation of its resistance to desiccation. Avian Pathol..

[28] Rothrock M.J., Al Hakeem W.G., Oladeinde A., Looft T., Li X., Guard J.Y. (2024). *Salmonella* biomapping of a commercial broiler hatchery. J. Food Prot..

[29] Cason J.A., Cox N.A., Bailey J.S. (1994). Transmission of *Salmonella typhimurium* during hatching of broiler chicks. Avian Dis..

[30] Bailey J.S., Cox N.A., Berrang M.E. (1994). Hatchery-acquired salmonellae in broiler chicks. Poult. Sci..

[31] Wang J., Fenster D.A., Vaddu S., Bhumanapalli S., Kataria J., Sidhu G., Leone C., Singh M., Dalloul R.A., Thippareddi H. (2024). Colonization, spread and persistence of *Salmonella* (Typhimurium, Infantis and Reading) in internal organs of broilers. Poult. Sci..

[32] Cox N.A., Berrang M.E., Cason J.A. (2000). *Salmonella* penetration of egg shells and proliferation in broiler hatching eggs—A review. Poult. Sci..

[33] Samberg Y., Meroz M. (1995). Application of disinfectants in poultry hatcheries. Rev. Sci. Et Tech. (Int. Off. Epizoot.).

[34] Bennett B. (2017). The importance of biosecurity in the modern day hatchery. Int. Hatch. Pract..

[35] Kustra K., Trela M., Hejdysz M., Kaczmarek S., Węsierska E., Babuszkiewicz M., Lis M.W. (2024). A conventional hatchery vs “on-farm” hatching of broiler chickens in terms of microbiological and microclimatic conditions. Animal.

[36] APHA (2024). Salmonella in Animals and Feed in Great Britain 2023.

[37] APHA (2023). Salmonella in Animals and Feed in Great Britain 2022.

[38] APHA (2019). Salmonella in Livestock Production in GB, 2018.

[39] EFSA, ECDC (2019). The European Union One Health 2018 Zoonoses Report. EFSA J..

[40] PHE (2018). Zoonoses Report UK 2017.

[41] Grimont P., Weill F. (2007). Antigenic Formulae of the Salmonella serovars.

[42] VMD (2018). UK Veterinary Antibiotic Resistance and Sales Surveillance, UK-VARSS, Supplementary Material.

[43] Hosmer D.W., Lemeshow S., Sturdivant R.X. (2013). Applied Logistic Regression.

[44] McFadden D., Hensher D.A., Stopher P.R. (1979). Quantitative methods for analysing travel behavior of individuals: Some recent developments. Behavioural Travel Modelling.

[45] McNemar Q. (1947). Note on the sampling error of the difference between correlated proportions or percentages. Psychometrika.

[46] Wilson E.B. (1927). Probable inference, the law of succession, and statistical inference. J. Am. Stat. Assoc..

[47] StataCorp (2017). Stata Statistical Software.

[48] Ha J.S., Seo K.W., Kim Y.B., Kang M.S., Song C.-S., Lee Y.J. (2018). Prevalence and characterization of *Salmonella* in two integrated broiler operations in Korea. Ir. Vet. J..

[49] Ren X., Li M., Xu C., Cui K., Feng Z., Fu Y., Zhang J., Liao M. (2016). Prevalence and molecular characterization of *Salmonella enterica* isolates throughout an integrated broiler supply chain in China. Epidemiol. Infect..

[50] Wu C., Deng Y., Chen Z., Peng J., Wu P., Chen J., Chen P., Liao M., Xu C., Zhang J. (2025). Prevalence, antimicrobial resistance and phylogenetic analysis of *Salmonella* contamination and transmission in yellow-feathered broiler hatcheries in China. Vet. Anim. Sci..

[51] Dunn A. (2026). Hatchery Salmonella Outbreak Raises Supply Concerns. Farmers Weekly.

[52] Corry J.E., Allen V.M., Hudson W.R., Breslin M.F., Davies R.H. (2002). Sources of *Salmonella* on broiler carcasses during transportation and processing: Modes of contamination and methods of control. J. Appl. Microbiol..

[53] Slader J., Domingue G., Jorgensen F., McAlpine K., Owen R.J., Bolton F.J., Humphrey T.J. (2002). Impact of transport crate reuse and of catching and processing on *Campylobacter* and *Salmonella* contamination of broiler chickens. Appl. Environ. Microbiol..

[54] Joseph B., Otta S.K., Karunasagar I., Karunasagar I. (2001). Biofilm formation by *Salmonella* spp. on food contact surfaces and their sensitivity to sanitizers. Int. J. Food Microbiol..

[55] Hussein A.G., Gamaleldin M.A. (2024). Evaluation of the tolerance of biofilm forming *Salmonella* isolated from dead in shell embryos to some disinfectants. Assiut Vet. Med. J..

[56] Ramesh N., Joseph S.W., Carr L.E., Douglass L.W., Wheaton F.W. (2002). Evaluation of chemical disinfectants for the elimination of *Salmonella* biofilms from poultry transport containers. Poult. Sci..

[57] Milanov D., Ljubojević D., Čabarkapa I., Karabasil N., Velhner M. (2017). Biofilm as risk factor for *Salmonella* contamination in various stages of poultry production. Eur. Poult. Sci..

[58] Gosling R.J., Mawhinney I., Vaughan K., Davies R.H., Smith R.P. (2017). Efficacy of disinfectants and detergents intended for a pig farm environment where *Salmonella* is present. Vet. Microbiol..

[59] Chojecka A., Tarka P., Kanecki K., Nitsch-Osuch A. (2019). Evaluation of the bactericidal activity of didecyl dimethyl ammonium chloride in 2-propanol against *Pseudomonas aeruginosa* strains with adaptive resistance to this active substance according to European standards. Tenside Surfact. Deterg..

[60] Aksoy A., El Kahlout K., Yardimci H. (2020). Comparative evaluation of the effects of binzalkonium chloride, iodine, gluteraldehyde and hydrogen peroxide disinfectants against avian salmonellae focusing on genotypic resistance pattern of the salmonellae serotypes toward benzalkonium chloride. Braz. J. Poult. Sci..

[61] Murtough S.M., Hiom S.J., Palmer M., Russell A.D. (2001). Biocide rotation in the healthcare setting: Is there a case for policy implementation?. J. Hosp. Infect..

[62] Russell A.D. (2002). Mechanisms of antimicrobial action of antiseptics and disinfectants: An increasingly important area of investigation. J. Antimicrob. Chemother..

[63] Griggs D.J., Gensberg K., Piddock L.J. (1996). Mutations in *gyrA* gene of quinolone-resistant *Salmonella* serotypes isolated from humans and animals. Antimicrob. Agents Chemother..

[64] Ryan M.P., Dillon C., Adley C.C. (2011). Nalidixic acid-resistant strains of *Salmonella* showing decreased susceptibility to fluoroquinolones in the midwestern region of the Republic of Ireland due to mutations in the *gyrA* gene. J. Clin. Microbiol..

[65] APHA (2020). Salmonella in Livestock Production in GB, 2019.

[66] Fernandez Marquez M.L., Burgos M.J., Pulido R.P., Galvez A., Lopez R.L. (2017). Biocide tolerance and antibiotic resistance in *Salmonella* isolates from hen eggshells. Foodborne Pathog. Dis..

[67] Jutkina J., Marathe N.P., Flach C.F., Larsson D.G.J. (2018). Antibiotics and common antibacterial biocides stimulate horizontal transfer of resistance at low concentrations. Sci. Total Environ..

[68] Maertens H., Demeyere K., De Reu K., Dewulf J., Vanhauteghem D., Van Coillie E., Meyer E. (2020). Effect of subinhibitory exposure to quaternary ammonium compounds on the ciprofloxacin susceptibility of *Escherichia coli* strains in animal husbandry. BMC Microbiol..

[69] Daehre K., Projahn M., Friese A., Semmler T., Guenther S., Roesler U.H. (2018). ESBL-producing *Klebsiella pneumoniae* in the broiler production chain and the first description of ST3128. Front. Microbiol..

[70] Projahn M., Daehre K., Semmler T., Guenther S., Roesler U., Friese A. (2018). Environmental adaptation and vertical dissemination of ESBL-/pAmpC-producing *Escherichia coli* in an integrated broiler production chain in the absence of an antibiotic treatment. Microb. Biotechnol..

[71] Wales A., Gosling R.J., Bare H.L., Davies R.H. (2021). Disinfectant testing for veterinary and agricultural applications: A review. Zoonoses Public Health.

[72] Maillard J.-Y., Pascoe M. (2024). Disinfectants and antiseptics: Mechanisms of action and resistance. Nat. Rev. Microbiol..

