# Supplementary material for: Salmonella enterica 13,23:i:- in a UK Broiler Hatchery: A Longitudinal Case Study of the Distribution of Resident Contamination and Its Control
Source: Microorganisms. 2026 Aug 8;14(8):1747. doi: 10.3390/microorganisms14081747 (PMC13515924; doi:10.3390/microorganisms14081747)
Supplement: Supplementary file 1 [file microorganisms-14-01747-s001.zip › microorganisms-4440307-supplementary.pdf]

Table S1: details of sampling locations and results

Area sampled	Item sampled	Result 13/08/19	Result 16/11/18	Result 18/07/18	Result 09/01/18	Result 10/10/17	Result 10/08/17	Result 06/05/15	Serotypes
**Egg Loading Bay**	floor	0/2	0/2	0/2	0/3	0/3	1/3	0/12	*S.* 13,23:i:-
	lorry	0/1	0/1	0/1					
	pallets/egg boxes etc.	-	-	-	0/1	0/2	0/2	0/14	
**Egg Reception**	floor	0/8	0/8	0/8	0/8	3/8	1/8	0/4	*S.* 13,23:i:-
	drains	0/1	0/1	0/1	0/1	0/3	0/3		
	egg trolleys	0/4	0/4	0/4	0/4	0/4	0/4	0/2	
	cleaning tools	0/1	0/1	0/1	0/1	0/1	1/1		*S.* 13,23:i:-
**Egg Store(s)**	floor	0/8	0/4	0/4	0/6	0/6	0/6	0/7	
	setter trolleys	-	-	-	0/3	0/4	0/4	0/6	
	doors	-	-	-	-	0/1	0/1		
	cleaning tools	0/1	0/1	0/1	0/1	0/1	0/1		
	Fumigation	0/1	-	-	-	-	-	0/3	
**Egg Tray Wash**	floor	0/6	1/7	1/7	1/4	-	-		*S.* 13,23:i:-

	equipment	0/4	3/8 (clean end trolley wash, liquid & solid egg waste containers	2/7_(belting & discharge chutes +ve)	0/8	-	-		*S.* 13,23:i:-
**Trolley wash**	floor/washer area	-	2/4 (drains)	0/4	1/3	-	-		*S.* 13,23:i:-
	Trolleys				0/3				
**Corridor/ Stairs**	floor	-	-	-	-	0/4	0/4		
**Paper Store**	floor	-	-	-	-	0/3	0/3		
	packed papers	0/2	-	-	-	0/3	0/3		
**Setter Area A**	floor	0/1	0/2	0/2	0/2	0/2	0/2	0/27*	
	drain	0/1	0/1	0/1	1/2	0/2	0/2		*S.* 13,23:i:-
	scraper	-	-	-	-	0/1	0/1		
	setter interiors	0/20	1/20 (setter 4)	0/20	4/20 (3 setters)	3/20 (2 setters)	1/20	0/84*	*S.* 13,23:i:-
**Corridor/ Stairs**	floor	-	-	-	0/2	1/2	0/2		*S.* 13,23:i:-
**Setter Area B**	floor	0/1	0/1	0/1	0/2	0/2	0/2		
	drain	0/1	0/1	0/1	0/2	0/2	0/2		
	setter trolleys/trays	-	-	-	-	0/4	0/4		
	setter interiors	0/8	0/8	0/8	0/8	0/8	1/8		*S.* 13,23:i:-

**Main Setter Corridor**	floor	-	0/2	0/2	0/2	0/4	0/4		
	drains	-	0/2	0/2	0/2	0/4	0/4		
	cleaning tools	0/1	0/1	0/1	0/1	0/1	1/1		*S.* 13,23:i:-
	clean hatcher baskets	0/8	0/8	0/8	0/4	0/2	0/2	0/8*	
	doors	-	-	-	-	0/2	0/2		
	floor scrub machine	-	0/1	0/1	0/1	0/1	0/1		
**Setter Area C**	floor	0/1	0/1	0/1	0/2	0/2	0/2		
	drains	0/1	0/1	0/1	0/2	0/2	0/2		
	setter interiors	0/20	0/20	0/20	1/20	3/20	1/20		*S.* 13,23:i:-
**Workshop**		-	-	-	0/2	-	-		
**Setter Area D**	floor	0/1	0/1	0/1	0/2	0/2	0/2		
	drains	0/1	0/1	0/1	0/2	0/2	1/2		*S.* 13,23:i:-
	clean hatcher baskets	-	-	-	1/2	0/2	0/2		
	setter interiors	0/12	1/12	0/12	1/12	1/12	2/12		*S.* 13,23:i:-
**Setter Area E**	floor	0/1	0/1	0/1	1/2	0/2	1/2		*S.* 13,23:i:-
	drains	0/1	0/1	0/1	0/2	0/2	0/2		

	setter interiors	0/27	0/28	0/28	4/28	0/28	4/28		*S.* 13,23:i:-
	Cleaning tools	0/2	-	-	-	-	-		
**Setter Area F**	floor	0/1	0/1	0/1	0/2	0/1	0/1		
	drain	0/1	0/1	0/1	0/2	0/1	0/1		
	setter interiors	0/12	0/12	0/12	0/12	0/12	0/12		
**Transfer Room**	floor	0/8	0/8 (in use)	0/8 (in use)	0/8	0/8	0/8	0/3	
	drains	0/2	0/2	0/2	0/3	0/3	1/3		*S.* 13,23:i:-
	transfer machine	0/9	0/9	0/9	1/9	0/9	0/9	0/2	
	back-up machine	-	-	-	-	0/2	0/2		
	clean hatcher baskets	-	-	-	-	0/2	0/2		
	cleaning tools	0/1	0/1	0/1	0/1	0/1	1/1		*S.* 13,23:i:-
**Electrical panel room**	floor	-	0/1	0/1	-	-	-	0/14	
	doors	-	0/1	0/1	-	0/1	0/1		
**Hatcher Room 1**	plenum corridor vents	0/2	0/2	0/2	1/2	0/2	0/2	0/16*	
	floor	0/2	0/2	0/2	0/2	1/2	0/2	0/12*	
	drains	0/1	0/1	0/1	0/2	0/2	2/2		*S.* 13,23:i:-

	hatcher interiors	0/12	2/12 (2 empty, cleaned hatchers)	1/12	2/12	0/12	5/12	0/60*	*S*. 13,23:i:-
**Hatcher Room 2**	floor	0/2	0/2	0/2 (hatch in 2 days)	0/2	2/2	0/2 (empty & cleaned first visit)		
	plenum corridor vents	0/2	-	0/2	1/2	0/2	0/2		*S*. 13,23:i:-
	drain	0/1	1/1	0/1	0/2	1/2	0/2		*S*. 13,23:i:-
	hatcher interiors	0/12 (chicks hatching)	9/12 (take-off next day)	0/12	7/8 (all except one hatcher +ve)	9/18 (all hatchers +ve, just cleaned)	0/18 (cleaned at first visit)		*S*. 13,23:i:-
**Hatcher room 3**	floor	0/2	0/1	0/2	0/2	0/2	0/2		
	drain	0/1	0/1	1/1	0/2	0/1	0/1		*S*. 13,23:i:-
	plenum vents	0/2	0/2	0/2	1/2	2/2	0/2		*S*. 13,23:i:-
	hatcher interiors	0/12 (eggs in yesterday, formalin in place)	0/12	0/12	0/12	4/12 (empty and cleaned)	0/12		*S*. 13,23:i:-
**Hatcher Room 4**	floor	0/2	0/2	0/2	0/2	2/2	0/2 (hatching in 3 days first visit)		*S*. 13,23:i:-
	drain	0/1	0/1	0/1	2/2	1/1	1/1		*S*. 13,23:i:-

	plenum	0/2	0/2	0/2	0/2	2/2	1/2		*S.* 13,23:i:-
	hatcher interiors	0/8	4/8 (4 hatchers; take-off in 4 days)	0/8 (take-off next day)	4/8 (3 hatchers)	6/8 (4 hatchers)	4/8 (3 hatchers)		*S.* 13,23:i:-
**Hatcher Room 6**	plenum vents	0/2	2/2	0/2	1/2	2/2	2/2		*S.* 13,23:i:-
	floor	0/1	0/2	0/2	0/2	-	-		
	drain	0/1	1/1	0/1	1/2	-	-		*S.* 13,23:i:-
	hatcher interiors	0/4	4/8 (4 hatchers – take off in 4 days)	0/8	5/8 (all hatchers)	2/4 (2 hatchers)	3/4 (3 hatchers – empty and cleaned)		*S.* 13,23:i:-
	vents/belt slots	-	-	-	-	-	3/4 (3 hatchers)		*S.* 13,23:i:-
**Hatcher Room 5**	floor	0/1	0/2	0/2	2/2	0/1	1/1		*S.* 13,23:i:-
	drain	0/1	0/1	0/1	0/2	0/1	1/1		*S.* 13,23:i:-
	plenum vents	0/2	0/2	0/2	0/2	0/1	1/2		*S.* 13,23:i:-
	hatcher interiors	0/8	2/8 (2 hatchers)	0/8	3/8 (2 hatchers)	1/6	6/8 (4 hatchers)		*S.* 13,23:i:-
**Hatchers**	Clean hatcher baskets							0/4	

**Chick Take-Off**	floor	0/4	2/4	0/4	2/4	4/4	3/4	0/5	*S.* 13,23:i:-
	drain	0/1	1/1	1/1	0/1	1/1	1/1		*S.* 13,23:i:-
	chick belt	0/4	4/4	0/4	1/4	4/4	4/4	0/6	*S.* 13,23:i:-
	macerator	0/4	4/4	1/4	3/4	3/4	3/4	2/4	*S.* 13,23:i:-
	floor beneath macerator	0/1	3/3	2/3	2/3	3/3	3/3		*S.* 13,23:i:-
	Waste from take-off	1/1	-	-	-	-	-		*S.* 13,23:i:-
**Chick Processing**	floor beneath chick line (sexing tables)	0/9	8/9	1/9	4/9	5/9	2/9	0/5	*S.* 13,23:i:-
	chick processing equipment	0/8	5/8	0/8	2/8	7/8	0/8	0/3	*S.* 13,23:i:-
	Cleaning tools	0/1	-	-	-	-	-	1/1	*S.* 13,23:i:-
**Chick Holding Area**	floor	0/8	2/8	0/8	2/4	2/2	1/2	0/2	*S.* 13,23:i:-
	drains	0/4	1/4	0/4	1/4	1/2	0/2		*S.* 13,23:i:-
	tables/belts	0/2	1/2	0/2	1/2	-	0/2		*S.* 13,23:i:-
**Loading Bay**	floor	0/5	1/2	0/2	0/2	0/1	1/1	0/4	*S.* 13,23:i:-
	Clean chick baskets	0/3	-	-	-	-	-		

	Occupied chick baskets	0/3	-	-	-	-	-	0/4	
**Hatcher Basket Wash Area**	washer interior: clean end	0/2	0/2	2/2	0/2	2/2	1/2		*S*. 13,23:i:-
	washer interior: dirty end	0/2	0/2	0/2	1/2	1/2	1/2		*S*. 13,23:i:-
	water reservoir tanks along machine	0/2	0/6	4/6	-	-	-		*S*. 13,23:i:-
	washer belt – far end / general	0/9	0/2	0/2	-	-	-	0/9	
	floor around washer	02	2/4	4/4	1/4	1/2	2/2	0/6	*S*. 13,23:i:-
	walls/ledges	0/2	0/4	1/4	1/4	-	-	0/6	*S*. 13,23:i:-
	dirty hatcher baskets	-	-	--	-	-	0/2		
	Drain (white crate wash room)							1/1	*S*. 13,23:i:-
**Hatcher Basket Store**	floor	0/6	5/6	1/6	0/3	-	-		*S*. 13,23:i:-
	clean hatcher baskets	0/7	0/6	0/6	0/5	0/4	3/4	0/7	*S*. 13,23:i:-

**Delivery Box Wash**	floor	0/4	0/4	0/4	0/4	-	-	0/4	
	washer	0/4	0/4	0/4	0/2	-	-	0/2	
	clean boxes	0/4	0/5	0/5	1/4	-	-	0/8	*S.* 13,23:i:-
	ledges	0/2	1/2	0/2	-	-	-		*S.* 13,23:i:-
	cleaning tools	0/2	0/2	0/2	-	-	-		
**Exterior**	waste skips	0/4	2/4	3/4	3/4	1/2	2/2		*S.* 13,23:i:-
	tanker	2/2	-	-	-	-	-		*S.* 13,23:i:-
	Small temporary waste tank	0/2	-	-	-	-	-		
	Skip							1/1	*S.* Give
	floor by skips	2/6	0/4	4/4	1/4	-	-	3/3	*S.* 13,23:i:-
	lorry interior	-	0/2	0/2	-	-	-	0/3	
	lorry standing/wash	3/6	3/7 (not wash area)	6/7	4/8	-	-		*S.* 13,23:i:-
**Area by back door**	floor	0/2							
	Wellies by back door	1/1	-	-	-	-	-		*S.* Idikan

*Totals for all setters or hatchers (not specified per room)

Table S2: Multivariable logistic regression analysis of factors ‘Location’ and ‘Visit number’ associated with the odds of a sample returning a positive *Salmonella* result

Variable	Odds Ratio	95% Confidence Interval	*p* Value
**Location category**			
Setters	*Reference*		
Chick Handling	15.497	(9.723 - 24.699)	<0.001
Tray Wash	8.694	(5.130 - 14.736)	<0.001
Egg Handling	0.562	(0.245 - 1.291)	0.175
Hatchers	7.662	(5.093 - 11.527)	<0.001
Miscellaneous	1.988	(0.792 - 4.991)	0.143
Waste / Outside	93.021	(51.149 - 169.172)	<0.001
**Visit number**			
1	1.666	(0.608 - 4.561)	0.321
2	31.774	(14.526 - 69.502)	<0.001
3	40.771	(18.625 - 89.247)	<0.001
4	20.052	(9.282 - 43.319)	<0.001
5	5.909	(2.662 - 13.116)	<0.001
6	19.019	(8.822 - 40.998)	<0.001
7	*Reference*		
